# Expression of the RANK/RANKL/OPG system in the human intervertebral disc: implication for the pathogenesis of intervertebral disc degeneration

**DOI:** 10.1186/s12891-019-2609-x

**Published:** 2019-05-17

**Authors:** Tomohiko Sano, Koji Akeda, Junichi Yamada, Norihiko Takegami, Takao Sudo, Akihiro Sudo

**Affiliations:** Department of Orthopaedic Surgery, Mie University Graduate School of Medicine, 2-174 Edobashi, Tsu City, Mie 514-8507 Japan

**Keywords:** Receptor activator of nuclear factor kappa B, Receptor activator of nuclear factor kappa B ligand, Osteoprotegerin, Intervertebral disc, Degeneration, Anti-receptor activator of nuclear factor kappa B ligand antibody, Proinflammatory cytokine, Matrix-degrading enzyme

## Abstract

**Background:**

The expression of the receptor activator of nuclear factor kappa B (RANK) /RANK ligand (RANKL) /osteoprotegerin (OPG) system and its association with the progression of intervertebral disc (IVD) degeneration has recently been reported in a human IVD. However, the effect of the RANK/RANKL/OPG system on the matrix metabolism of human IVD cells, especially on the expression of catabolic factors relevant to IVD degeneration, remains unknown. The purpose of this study was to examine the expression of the RANK/RANKL/OPG system, and then to evaluate the effect of this system on the expression of catabolic factors by human IVD cells.

**Methods:**

Annulus fibrosus (AF) and nucleus pulposus (NP) cells isolated by sequential enzyme digestion from human IVD tissues obtained during spine surgeries were monolayer cultured. The expression of the RANK/RANKL/OPG system was determined using immunohistochemical methods and real-time polymerase chain reaction (PCR). To evaluate the influence of interleukin-1 beta (IL-1β) stimulation on the mRNA expression of RANK, RANKL, and OPG, recombinant human IL-1β (rhIL-1β) was administered in the culture media of IVD cells. To examine the influence of RANKL signaling on the expression of matrix metalloprotease-3 (MMP-3), MMP-13, and IL-1β, the cells were cultured with exogenous recombinant human RANKL (rhRANKL), recombinant human OPG (rhOPG) or anti-human RANKL mouse monoclonal antibody (ahRANKL-mAB) with or without rhIL-1β.

**Results:**

Immunoreactivity to RANK/RANKL/OPG and the mRNA expression of the three genes were obviously identified in both AF and NP cells. rhIL-1β stimulation significantly upregulated the mRNA expression level of RANK/RANKL/OPG. The mRNA expression of catabolic factors was significantly upregulated by stimulation of rhRANKL in the presence of rhIL-1β. On the other hand, the administration of either rhOPG or ahRANKL-mAB significantly suppressed the mRNA expression of catabolic factors that had been upregulated by rhIL-1β stimulation. The suppressive effect of ahRANKL-mAB against rhIL-1β stimulation was also confirmed by the protein expression of MMP-3.

**Conclusions:**

The present study showed that the RANK/RANKL/OPG system may be involved in the progression of IVD degeneration. This study also suggested the potential use of anti-RANKL monoclonal antibody and OPG as therapeutic agents to suppress the progression of IVD degeneration.

## Background

The intervertebral disc (IVD) is composed of a central gelatinous nucleus pulposus (NP), a peripherally located annulus fibrosus (AF), and the cranial and caudal cartilaginous endplates associated with its capillary beds. In the degenerative IVD, alterations in cellular activity occur, changing the composition and concentration of extracellular matrix proteins: the synthesis of type II collagen and proteoglycans decreases and the synthesis of type I collagen increases, leading to tissue dehydration and fibrosis. Degenerative IVD cells upregulate tissue degradative enzymes, such as the matrix metalloproteinases (MMPs) and a disintegrin and metalloproteinase with thrombospondin motifs (ADAMTSs), which play a role in matrix degradation during disc degeneration [1–3]. Interleukin-1β (IL-1β) and tissue necrosis factor-α (TNF-α) are considered to strongly influence these degenerative processes [4]. Proinflammatory cytokines, including IL-1β and TNF-α, are overexpressed in degenerated and herniated IVDs [5, 6], resulting in the loss of tissue cellularity by upregulation of genes involved with the apoptotic pathway [7, 8]. Proinflammatory cytokines also stimulate the expression of matrix-degrading enzymes and decrease the synthesis of matrix proteins, consequently leading to homeostatic imbalance, followed by disruption of the extracellular matrix that characterizes IVD degeneration (see review in [8]). These biochemical and molecular changes within the degenerated IVD are considered to be associated with low back pain and degenerative disc diseases [9, 10].

The receptor activator of nuclear factor kappa B (NF-κB) ligand (RANKL), initially identified as a member of the TNF ligand superfamily, is well known to regulate bone metabolism [11, 12]. Previous studies have shown that the signal from RANKL and its receptor (RANK) play an essential role in the differentiation, activity and survival of osteoclasts [10]. RANKL interacts with RANK, which is expressed on the membranes of mature osteoclasts and osteoclast precursors, to promote differentiation and activation of osteoclasts. Osteoprotegerin (OPG), which is secreted by stromal cells and osteoblasts, acts as a soluble decoy receptor for RANKL. By binding to RANKL, OPG inhibits the interaction of RANKL-RANK, thereby preventing RANK activation and subsequent osteoclastogenesis [11, 12].

The RANK/RANKL/OPG system has been shown to be expressed in human articular cartilage; however, its functional role and relevance to the pathogenesis of knee osteoarthritis remains unknown [13, 14]. On the other hand, RANKL and OPG have also been reported to be expressed by the human IVD and are considered to be involved with the degeneration process of IVDs [15–18]. We previously found the expression of the RANK/RANKL/OPG system in the rat IVD. In the rat IVD, the expression of RANKL is regulated by stimulation with IL-1β and the expression of catabolic factors, such as IL-1β, MMP-3, and MMP-13, is enhanced by stimulation with RANKL in the presence of IL-1β [18]. Therefore, we hypothesized that the RANK/RANKL/OPG system may play a part in the complex molecular mechanism of human IVD degeneration by interactions with the proinflammatory cytokines network, and that this system would be involved in the degenerative process of the human IVD.

The purposes of this study were (1) to examine the expression of the RANK/RANKL/OPG system by human IVD cells with or without pro-inflammatory stimulation, and (2) to evaluate the effects of the RANK/RANKL/OPG system on the expression of catabolic factors, including proinflammatory cytokines and matrix-degrading enzymes, by human IVD cells under stimulation by pro-inflammatory cytokine (IL-1β).

## Methods

### Human IVD tissues

Institutional Review Board (IRB) approval was obtained for this study. Written informed consent was obtained from all patients. Human IVD tissues obtained from spine surgeries were used in this study with 24 donors (average patient age: 64.1 ± 15.9 years-old) and 49 discs (Pfirrmann’s magnetic resonance imaging (MRI) [18] grade: grade 2 (*n* = 7); grade 3 (n = 7); grade 4 (*n* = 32); grade 5 (n = 3), average Pfirrmann grade: 3.6 ± 0.8) (Table. 1). All disc tissues from each donor were cultured at the same time.

**Table 1:** Information of donors and intervertebral discs used in this study

Patient #	Age	Gender	Number of IVDs	MRI Grade (disc level)	Experimental Protocol #
1	35–39	Male	2	2 (L1/2, L2/3)	1, 2, 5
2	35–39	Male	2	2 (T12/L1, L1/2)	1, 2
3	65–69	Male	2	4 (L3/4, L4/5)	1, 2
4	75–79	Female	1	5 (L3/4)	1
5	65–69	Male	2	4 (L3/4, L4/5)	1
6	25–29	Male	2	2 (L4/5, L5/S1)	2
7	65–69	Female	4	4 (L2/3, L4/5), 5 (L3/4), 3 (L5/S1)	2
8	80–84	Female	1	4 (L4/5)	3
9	70–74	Female	4	4 (L1/2-L4/5)	3
10	75–79	Female	3	4 (L2/3-L4/5)	3
11	65–69	Male	1	4 (L4/5)	3
12	75–79	Female	2	3 (L1/2), 4 (L4/5)	3
13	80–84	Female	2	4 (L3/4, L4/5)	4
14	70–74	Female	2	4 (T12/L1, L1/2)	4
15	50–54	Male	1	4 (L4/5)	4
16	65–69	Female	4	4 (L2/3-L5/S1)	4
17	75–79	Female	2	4 (L4/5, L5/S1)	4
18	70–74	Female	1	4 (L4/5)	4
19	75–79	Female	2	4 (L3/4, L4/5)	1, 4
20	40–44	Male	1	2 (L4/5)	4
21	55–59	Male	3	3 (L2/3, L3/4), 4 (L4/5)	4
22	65–69	Female	2	3 (L3/4), 4 (L4/5)	4
23	65–69	Male	1	4 (L5/S1)	4
24	80–84	Female	2	4 (L3/4, L4/5)	4

Experimental protocols: 1. Effect of IL-1β stimulation on mRNA levels of RANK/RANKL/OPG; 2. Effect of RANKL on the expression of proinflammatory cytokines and matrix-degrading enzymes with or without IL-1β stimulation; 3. Effect of OPG on the expression of proinflammatory cytokines and matrix-degrading enzymes with or without IL-1β stimulation; 4. Effect of anti-human RANKL antibody on the expression of proinflammatory cytokines and matrix-degrading enzymes with or without IL-1β stimulation; and 5. Immunofluorescent analysis of human IVD cells

### Cell culture

AF and NP cells were separately isolated by sequential enzyme digestion, and cultured in monolayer as previously reported [18]. In short, following 0.4% Pronase and 0.025% Collagenase P digestion, the cells were washed with Dulbecco’s modified Eagle’s medium and Ham’s F-12 medium (DMEM/F12; Gibco, Palo Alto, CA, USA), and cultured in monolayer at 4.0 × 10^4^ cells/mL with 5% CO2, 95% air in complete medium (DMEM/F12 containing 10% fetal bovine serum (FBS), 25 μg/mL ascorbic acid, and 50 μg/mL gentamicin). The medium was changed every third day. Primary cultured cells were used in all the experiments conducted in this study. The cells in 6-well plates were pre-cultured up to 80% confluency (approximately 7 days for AF cells or 14 days for NP cells) in all sets of experiments.

### Experimental protocol for culture conditions

#### Effect of IL-1β stimulation on mRNA levels of *RANK/RANKL/OPG*

Six donors (average age: 59.8 ± 18.8 (34–76) years-old, average Pfirrmann grade: 3.4 ± 1.1 (grade 2–5)) were used in this study (Table. 1). AF and NP cells were cultured in serum-free medium for 24 h for serum-starvation. The cells were then cultured in DMEM/F12 containing 0.3% FBS for an additional 24 h with or without recombinant human IL-1β (rhIL-β; R&D Systems, Minneapolis, MN, USA) at 0.1, 1.0, or 10 ng/ml.

#### Effect of RANKL on the expression of proinflammatory cytokines and matrix-degrading enzymes with or without IL-1β stimulation

Five donors (average age: 46.6 ± 20.1 (25–69) years-old, average Pfirrmann grade: 3.7 ± 1.1 (2–5)) were used in this study (Table. 1). Following serum-starvation, the cells were then cultured in DMEM/F12 containing 0.3% FBS for an additional 48 h with or without 10 ng/ml recombinant human RANKL (rhRANKL; Wako Pure Chemical, Osaka, Japan) in the presence or absence of rhIL-1β (1.0 ng/ml).

#### Effect of OPG on the expression of proinflammatory cytokines and matrix-degrading enzymes with or without IL-1β stimulation

Five donors (average age: 74.6 ± 6.7 (66–83) years-old, average Pfirrmann grade: 3.9 ± 0.3 (3–4)) were used in this study (Table. 1). Following serum-starvation, the cells were then cultured in DMEM/F12 containing 0.3% FBS for an additional 48 h with or without recombinant human OPG (rhOPG; Oriental Yeast, Tokyo, Japan) at 0.1 or 1.0 μg/ml in the presence or absence of rhIL-1β (1.0 ng/ml).

#### Effect of anti-human RANKL antibody on the expression of proinflammatory cytokines and matrix-degrading enzymes with or without IL-1β stimulation

Twelve donors (average age 67.6 ± 12.4 (43–84) years-old, average Pfirrmann grade: 3.8 ± 0.5 (3–4)) were used in this study (Table. 1). Among them, the AF tissues were used from 8 donors, and NP tissues from 9 donors. Following serum-starvation, the cells were then cultured in DMEM/F12 containing 0.3% FBS for an additional 48 h with or without anti-human RANKL mouse monoclonal antibody (ahRANKL-mAB; Oriental Yeast, Tokyo, Japan) at 0.1 or 1.0 μg/ml in the presence or absence of rhIL-1β (1.0 ng/ml).

### RNA isolation and quantitative real-time polymerase chain reaction (PCR)

Total cellular RNA was extracted from human AF and NP monolayer-cultured cells and processed for the synthesis of Complementary DNA (cDNA) as previously reported [18]. The sample products were stored at − 80 °C until analysis.

Following treatment with IL-1β and/or RANKL, OPG, or anti-human RANKL antibody, the resultant cDNA was amplified for the following target genes: RANK, RANKL, OPG, MMP-3, MMP-13, and IL-1β. Inventoried (ready-made) primers corresponding to target genes were used in this study (Table 2: TaqMan Gene Expression Assays, Applied Biosystems). Real time PCR was performed using the ABI PRISM 7000 Sequence Detection System (Applied Biosystems) as previously reported [18]. The assay was calibrated using 18S ribosomal RNA (rRNA) as an internal control. The data were normalized by the relative expression of the control group in each set of experiments.

**Table 2:** Primers for real-time polymerase chain reaction (PCR)

Genes	Assay ID^a^	Size (bp)
*RANK*	Hs00921372_m1	**75**
*RANKL*	Hs00243522_m1	67
*OPG*	Hs00900358_m1	74
*IL-1β*	Hs01555410_m1	91
*MMP-3*	Hs00968305_m1	78
*MMP-13*	Hs00233992_m1	75
*18S rRNA*	Hs99999901_s1	187

RANK receptor activator of nuclear factor kappa B, RANKL rank ligand, OPG osteoprotegerin, IL interleukin, MMP matrix metalloproteinase, rRNA ribosomal RNA. ^a^TaqMan Gene Expression Assays (Applied Biosystems)

### Enzyme-linked immunosorbent assay

Following treatment with IL-1β and/or anti-human RANKL antibody, the concentration of MMP-3 in the culture medium was evaluated using an enzyme-linked immunosorbent assay (ELISA) kit (R&D Systems) according to the manufacturer’s instruction. The samples were stored at − 80 °C until analysis. Evaluations were done in duplicate. To adapt the MMP-3 concentration to the detectable range, the samples with IL-1β treatment were diluted 1000-fold, and the others were diluted 10-fold. Samples and standards were placed in microwells of a microwell plate with a diluent and incubated for 2 h at room temperature. The plate was then washed with washing buffer three times. Human MMP-3 conjugate was added to each well and incubated for 2 h at room temperature. Following washing with washing buffer three times, substrate solution was added to each well and incubated for 30 min at room temperature, protected from intense light. Stop solution was then added to each well and the absorbance of each well was read at 450 nm within 30 min.

### Immunofluorescent analysis of human IVD cells

Human AF and NP cells were monolayer-cultured on 4-chamber slides for 4 days, and processed for the immunofluorescent analysis as previously reported [18]. In short, the cells were incubated with a mouse monoclonal antibody for RANK (ab12008, 1:500; Abcam, Cambridge, UK), a rabbit polyclonal antibody for RANKL (ab9957, 1:500; Abcam), or a goat polyclonal antibody for OPG (sc-8468, 1:50; Santa Cruz Biotechnology, Santa Cruz, CA, USA) overnight at room temperature. For isotype control, the cells were incubated with mouse isotype-matched immunoglobulin (IgG; Dako, Glostrup, Denmark) instead of the primary antibodies. Secondary antibodies [Alexa 488 conjugated anti-mouse IgG (1:400; Molecular Probes, Eugene, OR, USA), or anti-rabbit IgG or anti-goat IgG] were applied for 3 h at room temperature. Nuclei were stained with propidium iodide (1:100; Molecular Probes) for 5 min. The slides were then cover-slipped with Vectashield mounting medium (Vector Laboratories, Burlingame, CA, USA). Samples were imaged using a confocal laser scanning microscopy (Fluoview FV1000; Olympus, Tokyo, Japan).

### Statistical analysis

The data are expressed as the mean ± standard error. The number of donors used in each study was described above (Table 1). The data were analyzed by one-way analysis of variance (ANOVA) using between-subject factors for the different experimental groups. The post hoc analyses were performed using the Fisher protected least significant differences test. The evaluation of statistical differences between the groups was determined using the Mann-Whitney U test. Significance was accepted at *p* < 0.05. All statistical analyses were performed using IBM SPSS Statistics (IBM Japan, Tokyo).

## Results

### Immunofluorescent analysis of human IVD cells

**Fig. 1:**
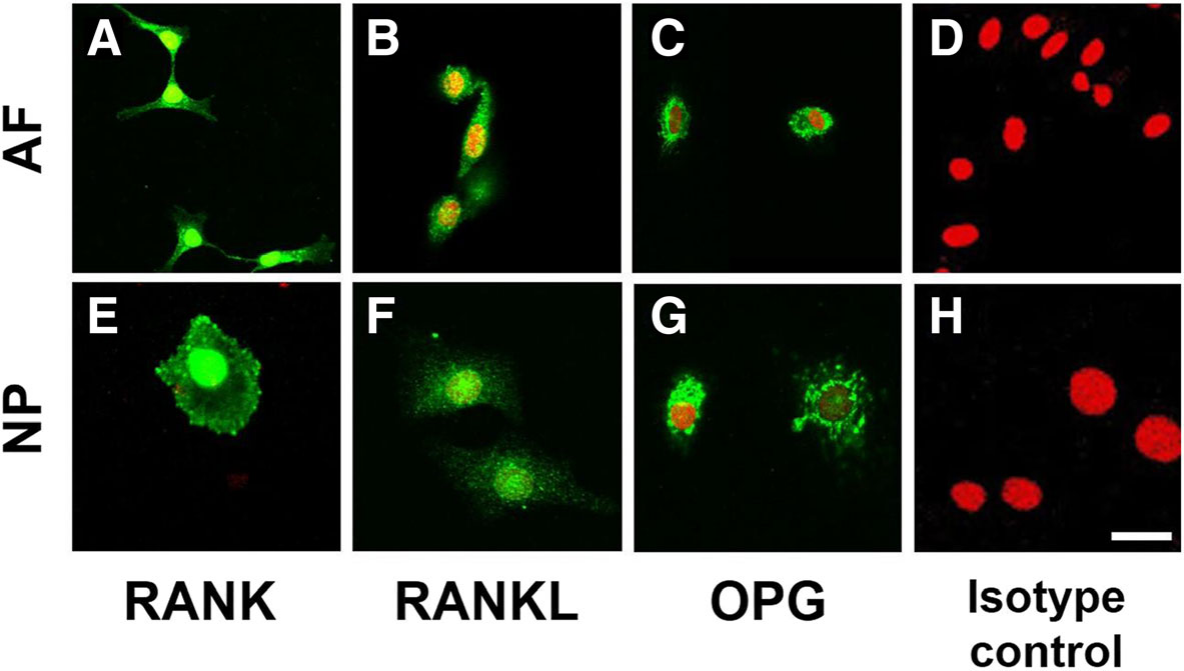
Immunohistochemical staining for receptor activator of nuclear factor kappa B (*RANK*)/RANK ligand (*RANKL*)/osteoprotegerin (*OPG*) in cultured annulus fibrosus (*AF*) and nucleus pulposus (*NP*) cells. All the cells were cultured in monolayer for 4 days: **a-d** AF; **e-h** NP. **d**, **h** Isotype controls. Samples were imaged using confocal microscopy. Immunoreactivity (*green*) is clearly seen in the AF, NP cells. Nuclei are stained with propidium iodide (*red*). *Scale bar* 10 μm

Fluorescent-immunoreactivity to RANK, RANKL and OPG was clearly identified in AF and NP monolayer-cultured cells (Fig. 1). Analysis by confocal microscopy showed that immunoreactivity to RANK was mainly found in cell membranes and cytoplasm of both AF and NP cells (Fig. 1a, e). RANKL-immunoreactivity was uniformly distributed in the cytoplasm of both AF and NP cells (Fig. 1b, f). A spot-like immunoreactivity of OPG was identified in the cytoplasm of both AF and NP cells (Fig. 1c, g). No immunoreactivity was found in the isotype controls (Fig. 1d, h).

### mRNA expression of *RANK*, *RANKL* and *OPG* in cultured human IVD cells

**Fig. 2:**
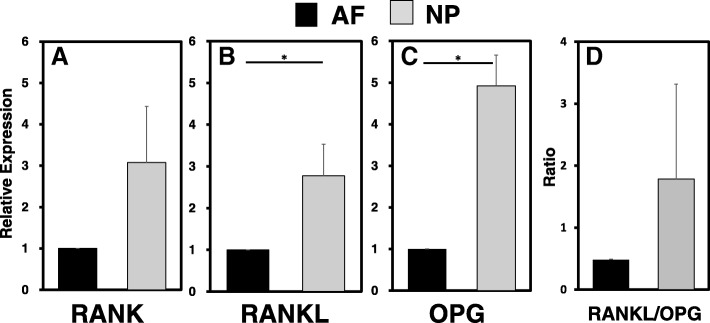
Detection of basal mRNA expressions of receptor activator of nuclear factor kappa B (*RANK*)/ RANK ligand (*RANKL*)/ osteoprotegerin (*OPG*) in annulus fibrosus (*AF)*, and nucleus pulposus (*NP*) cells. The mRNA expressions of *RANK* (a), *RANKL* (b), and *OPG* (c) were quantified by real-time polymerase chain reaction. Their expressions in NP cells were normalized by those in AF cells. Significantly higher mRNA expression levels of *RANKL* and *OPG* were found in NP cells than those in AF cell; *p < 0.05, **p < 0.01. There was no significant difference in the expression level of RANK between AF and NP cells. The ratio of *RANKL*/*OPG* (d) in NP cells had a tendency to be higher compared to that in AF cells

The qualitative and quantitative assessment of the expression of RANK, RANKL, and OPG were quantified using real-time PCR. Detectable level of mRNA expression of *RANK*, *RANKL*, and *OPG* were clearly identified in both AF and NP cells (Fig. 2). Although the mRNA expression of *RANK* by NP cells was higher than that of AF cells, there was no significant difference (relative expression in the NP (vs. AF): *RANK* 3.07 ± 0.92, n.s.) (Fig. 2a). The mRNA expression levels of *RANKL* and *OPG* by NP cells were significantly higher than those by AF cells (relative expression in the NP (vs. AF): *RANKL* 2.77 ± 0.76; *OPG* 4.92 ± 0.74, *p* < 0.05, respectively) (Fig. 2b, c). There were no significant differences in the ratio of RANKL/OPG between NP and AF cells (Fig. 2d).

### Effect of IL-1β treatment on mRNA levels of *RANK*, *RANKL* and *OPG*

**Fig. 3:**
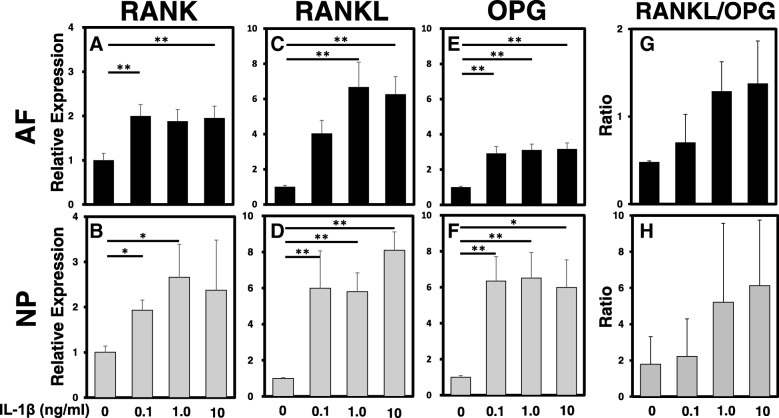
The effect of interleukin-1 beta (IL-1β) on mRNA levels of receptor activator of nuclear factor kappa B (*RANK*)/ RANK ligand (*RANKL*)/ osteoprotegerin (*OPG*) in annulus fibrosus (*AF*) and nucleus pulposus (*NP*) cells. The mRNA expressions of *RANK* (**a**, **b**), *RANKL* (**c**, **d**), and, *OPG* (**e**, **f**) by AF (a, c, e) and NP (**b**, **d**, **f**) cells were quantified by real-time polymerase chain reaction. Stimulation with IL-1β significantly increased mRNA expression of *RANK*, *RANKL*, and *OPG* by both AF and NP cells. There was a tendency that the ratio of RANKL/OPG by both AF and NP cells (**g**, **h**) was increased by stimulation with IL-1β: *p < 0.05, **p < 0.01 vs. control

IL-1β stimulation induced a significant but mild increase of mRNA expression of RANK in AF and NP cells (relative expression (vs. control): IL-1β 0.1 ng/mL: AF 2.0 ± 0.26, *p* < 0.01, NP 1.93 ± 0.23, *p* < 0.05; IL-1β 1.0 ng/mL: NP 2.66 ± 0.73, *p* < 0.05; IL-1β 10 ng/mL: AF 1.95 ± 0.27, *p* < 0.01) (Fig. 3a, b).

The mRNA expression levels of RANKL in both AF and NP cells were significantly upregulated by IL-1β (1.0 ng/mL) (relative expression (vs. control): AF 6.68 ± 1.41; NP 5.80 ± 1.04; *p* < 0.01, respectively), and by IL-1β (10 ng/mL) (relative expression (vs. control): AF 6.26 ± 1.00; NP 8.10 ± 1.02; *p* < 0.01, respectively) (Fig. 3c, d).

The mRNA expression levels of OPG in both AF and NP cells were significantly upregulated by treatment with IL-1β at 0.1 ng/mL (relative expression (vs. control): AF 2.92 ± 0.39; NP 6.35 ± 1.36; *p* < 0.01, respectively), at 1.0 ng/mL (relative expression (vs. control): AF 3.11 ± 0.34; NP 6.52 ± 1.42; *p* < 0.01, respectively), and at 10 ng/mL (relative expression (vs. control): AF 3.17 ± 0.34, *p* < 0.01; NP 6.00 ± 1.53, *p* < 0.05) (Fig. 3e, f). There were no significant changes in the ratio of RANKL/OPG in the presence or absence of IL-1β stimulation by both AF and NP cells (Fig. 3g, h).

### Effect of RANKL on the mRNA expression levels of proinflammatory cytokines and matrix-degrading enzymes

**Fig. 4:**
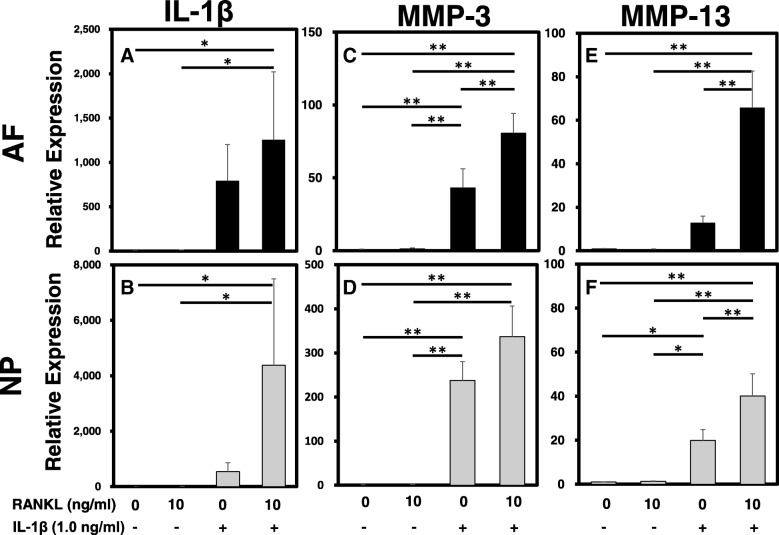
The effect of receptor activator of nuclear factor kappa B ligand (RANKL) on the mRNA expression of matrix-degrading enzymes and proinflammatory cytokines with or without recombinant human interleukin-1 beta (rhIL-1β) stimulation. The mRNA expression of *IL-1β* (a, b), matrix metalloprotease (*MMP*)*-3* (c, d), and *MMP-13* (e, f) by annulus fibrosus (AF) (a, c, e) and nucleus pulposus (NP) (b, d, f) cells were quantified by real-time polymerase chain reaction. The mRNA expression levels of *MMP-3* by AF cells and those of *MMP-13* by both AF and NP cells were significantly upregulated by stimulation of rhRANKL with rhIL-1β (1.0 ng/mL). Similar, but not significant trends were also identified in the expression level of *IL-1β* by both AF and NP cells, and that of *MMP-3* by NP cells: *p < 0.05, **p < 0.01

The mRNA expression levels of IL-1β, MMP-3, and MMP-13 in AF and NP cells were quantified using real-time PCR (Fig. 4). Treatment with RANKL alone did not induce a significant change in the expression of IL-1β (Fig. 4a, b), MMP-3 (Fig. 4c, d), and MMP-13 (Fig. 4e, f) by both AF and NP cells. However, stimulation with IL-1β significantly upregulated the mRNA expression of MMP-3 (Fig. 4c, d) in both AF and NP cells and that of MMP-13 in NP cells (Fig. 4f) (relative expression: RANKL 0 μg/ml + IL-1β (vs. control): AF: *MMP-3* 43.34 ± 12.95, *p* < 0.05; NP: *MMP-3*237.47 ± 42.88, *p* < 0.01; *MMP-13* 19.91 ± 4.88, *p* < 0.05). The mRNA expression levels of MMP-3 in AF cells and those of MMP-13 in both AF and NP cells were significantly further upregulated by stimulation of RANKL with rhIL-1β (1.0 ng/mL) compared to those of rhIL-1β only (relative expression: RANKL 10 ng/mL + IL-1β (vs. RANKL 0 ng/mL + IL-1β), AF: *MMP-3* 2.75 ± 0.38; *MMP-13* 6.20 ± 1.82; NP: *MMP-13* 1.69 ± 0.35; *p* < 0.01, respectively). There were no significant differences in the expression levels of IL-1β in both AF and NP cells, and those of MMP-3 in NP cells.

### Effect of OPG on the expression of proinflammatory cytokines and matrix-degrading enzymes

**Fig. 5:**
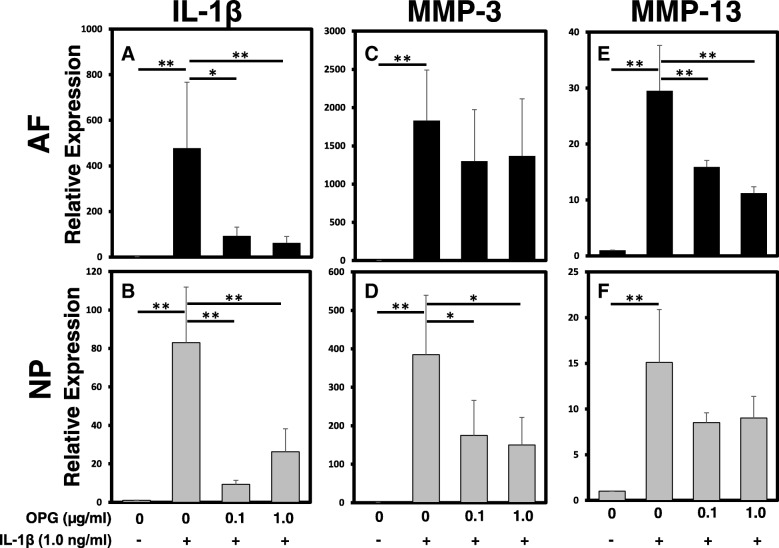
The effect of osteoprotegerin (OPG) on the mRNA expression of matrix-degrading enzymes and proinflammatory cytokines with or without recombinant human interleukin-1 beta (rhIL-1β) stimulation. The mRNA expression of *IL-1β* (**a**, **b**), matrix metalloprotease (*MMP*)*-3* (**c**, **d**), and *MMP-13* (e, f) by annulus fibrosus (AF) (**a**, **c**, **e**) and nucleus pulposus (NP) (**b**, **d**, **f**) cells were quantified by real-time polymerase chain reaction. Treatment with OPG in the presence of rhIL-1β (1.0 ng/mL) significantly downregulated the mRNA expression levels of *IL-1β* by both AF and NP cells*, MMP-3* by NP cells and *MMP-13* by AF cells. There was a similar trend in the expression levels of *MMP-3* by AF cells and that of *MMP-13* by NP cells: *p < 0.05, **p < 0.01

The mRNA expression levels of IL-1β, MMP-3, and MMP-13 by AF and NP cells were quantified using real-time PCR (Fig. 5). Treatment with IL-1β at 1.0 ng/ml significantly upregulated the mRNA expression levels of both IL-1β (Fig. 5a, b), MMP-3 (Fig. 5c, d), and MMP-13 (Fig. 5e, f) by both AF and NP cells (relative expression: OPG 0 μg/ml + IL-1β (vs. control): AF: *IL-1β* 477.75 ± 289.11; *MMP-3* 1829.59 ± 661.95; *MMP-13* 29.53 ± 8.08; NP: *IL-1β* 83.00 ± 28.94; *MMP-3* 384.92 ± 154.13; *MMP-13* 15.08 ± 5.80, *p* < 0.01, respectively). Treatment with OPG alone did not induce a significant effect on the mRNA expression of IL-1β, MMP-3, and MMP-13 by both AF and NP cells. However, OPG significantly suppressed the expression of IL-1β by AF and NP cells (Fig. 5a, b), MMP-3 by NP cells (Fig. 5d), and MMP-13 by AF cells (Fig. 5e) upregulated by IL-1β (1.0 ng/mL) stimulation (relative expression: OPG 1.0 μg/mL + IL-1β (vs. OPG 0 μg/mL + IL-1β), AF: *IL-1β* 0.77 ± 0.29; *MMP-13* 0.77 ± 0.22; *p* < 0.01, respectively, NP: *IL-1β* 0.62 ± 0.19, *p* < 0.01; *MMP-3* 0.81 ± 0.26, *p* < 0.05). There were no significant differences in the expression levels of MMP-3 by AF cells and those of MMP-13 by NP cells.

### Effect of anti-human RANKL antibody on the expression of proinflammatory cytokines and matrix-degrading enzymes

**Fig. 6:**
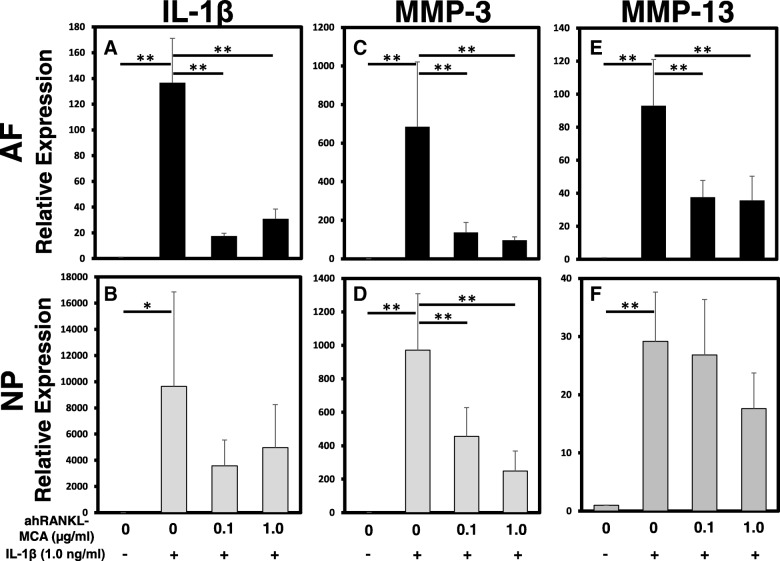
The effect of anti-human receptor activator of nuclear factor kappa B ligand mouse monoclonal antibody (ahRANKL-MCA) on the mRNA expression of matrix-degrading enzymes and proinflammatory cytokines with or without recombinant human interleukin-1 beta (rhIL-1β) stimulation. The mRNA expression of *IL-1β* (**a**, **b**), matrix metalloprotease (*MMP*)*-3* (**c**, **d**), and *MMP-13* (**e**, **f**) by annulus fibrosus (AF) (**a**, **c**, **e**) and nucleus pulposus (NP) (**b**, **d**, **f**) cells were quantified by real-time polymerase chain reaction. ahRANKL-MCA significantly suppressed the expression of *IL-1β, MMP-3 and MMP-13* by AF cells and that of *MMP-3* by NP cells upregulated by rhIL-1β (1.0 ng/mL) stimulation. There was a similar trend in the expression levels of *IL-1β* and *MMP-13* by NP cells: *p < 0.05, **p < 0.01

The mRNA expression levels of IL-1β, MMP-3, and MMP-13 by AF and NP cells were quantified using real-time PCR (Fig. 6). Treatment with IL-1β at 1.0 ng/ml significantly upregulated the expression levels of IL-1β, MMP-3, and MMP-13 by both AF and NP cells (relative expression: ahRANKL-mAB 0 μg/ml + IL-1β (vs. control): AF: *IL-1β* 136.72 ± 34.42; *MMP-3* 684.99 ± 335.81; *MMP-13* 92.98 ± 28.04; *p* < 0.01, respectively, NP: *IL-1β* 9647.31 ± 7213.02, *p* < 0.05; *MMP-3* 971.23 ± 336.88, *p* < 0.01; *MMP-13* 29.17 ± 8.46, *p* < 0.01) (Fig. 6a-f). Treatment by ahRANKL-mAB alone did not induce a significant change in the mRNA expression of IL-1β, MMP-3, and MMP-13 by both AF and NP cells. However, ahRANKL-mAB treatment significantly suppressed the expression of IL-1β (Fig. 6a) and MMP-13 (Fig. 6e) by AF cells and that of *MMP-3* by both AF and NP cells (Fig. 6c, d) upregulated by IL-1β (1.0 ng/mL) stimulation (relative expression: ahRANKL-mAB 1.0 μg/mL + IL-1β (vs. ahRANKL-mAB 0 μg/mL + IL-1β): AF: *IL-1β* 0.32 ± 0.10; *MMP-3* 0.37 ± 0.13; *MMP-13* 0.46 ± 0.13; NP: *MMP-3* 0.36 ± 0.06; *p* < 0.01, respectively). There were no significant differences in the mRNA expression levels of IL-1β and MMP-13 by NP cells.

**Fig. 7:**
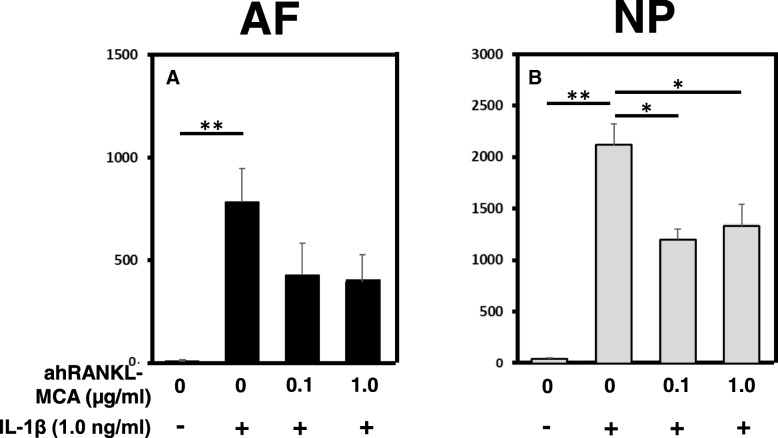
The effect of anti-human receptor activator of nuclear factor kappa B ligand mouse monoclonal antibody (ahRANKL-MCA) on the protein level of matrix metalloprotease (*MMP*)*-3* in the culture medium of cells with or without recombinant human interleukin-1 beta (rhIL-1β) stimulation. The protein level of *MMP-3* in the culture medium of annulus fibrosus (AF) and nucleus pulposus (NP) cells was quantified by an enzyme-linked immunosorbent assay (ELISA). Treatment with IL-1β (1.0 ng/mL) significantly upregulated the protein expression of MMP-3 by both AF and NP cells. ahRANKL-mAB significantly reduced the protein level of MMP-3 in the culture media of NP cells upregulated by IL-1β stimulation (b). In AF cells, a similar, but not significant, trend was identified (a)

The protein level of MMP-3 in the culture medium of AF and NP cells was evaluated using an ELISA kit (Fig. 7). Treatment with IL-1β (1.0 ng/mL) significantly upregulated the protein expression of MMP-3 by both AF and NP cells. Although treatment with ahRANKL-mAB alone did not induce significant changes in the concentration of MMP-3 in the culture medium, ahRANKL-mAB significantly reduced the protein level of MMP-3 in the culture media of NP cells upregulated by IL-1β stimulation (Fig. 7b). In AF cells, a similar, but not significant, trend was identified (Fig. 7a).

## Discussion

In this study, the constitutive expression of RANK, RANKL and OPG by human AF and NP cells was identified. In particular, the basal expression levels of the three molecules in NP cells were higher than those in AF cells. Stimulation with exogenous IL-1β remarkably upregulated the expression of RANK, RANKL and OPG by both AF and NP cells. The administration of exogenous RANKL alone did not induce a change in the expression of catabolic factors, including proinflammatory cytokines (e.g., IL-1β) and matrix-degrading enzymes (e.g., MMP-3 and -13) by human AF and NP cells. Interestingly, these catabolic factors were however significantly upregulated by treatment with RANKL in the presence of IL-1β. On the other hand, the administration of rhOPG in the presence of IL-1β suppressed the expression of catabolic factors by human AF and NP cells upregulated by IL-1β stimulation. A similar effect was identified by treatment with anti-human RANKL monoclonal antibody (ahRANKL-mAB) in the presence of IL-1β.

Our quantitative mRNA analysis showed that the constitutive expression of the RANK/RANKL/OPG system in NP cells was higher than that in AF cells. The NP in the adult human IVD, originally derived from the central notochord in the embryo, is rich in chondrocyte-like cells, whereas the AF, derived from mesenchymal tissue surrounding the central notochord, is rich in fibrocartilage-like cells [1, 19]. The biological difference between NP and AF cells might account for the differences in the constitutive expression of the RANK/RANKL/OPG system. Byron et al. reported that a high concentration of OPG was found in the culture media of equine articular chondrocytes compared to that of equine articular synovial fibroblasts [20], suggesting that the production of OPG was higher in a chondrocytic phenotype than in a fibroblastic phenotype. Similar to the results of Byron’s study, the results of this study suggest that the basal metabolism activity of the RANK/RANKL/OPG system in NP cells is higher than that in AF cells.

To imitate the micro-environment in degenerated IVDs in which proinflammatory cytokines were overexpressed [5], in this study, human IVD cells were cultured in the presence of proinflammatory cytokine IL-1β. Stimulation with IL-1β significantly upregulated the mRNA expressions of RANK/RANKL/OPG. The expression of RANKL and OPG was more upregulated by IL-1β stimulation than that of RANK. Similar findings on the stimulative effects of IL-1β have been reported for osteoblasts [21–23]; the imbalance among RANK/RANKL/OPG expressions is considered to be associated with the progression of inflammatory osteolytic diseases [24]. Therefore, we speculated that the imbalance of these molecules by human IVD cells might be related to the homeostatic imbalance that is found in the pathogenesis of human IVD degeneration.

Our previous study evaluated the expression of the RANK/RANKL/OPG system in human IVD tissues using immunohistochemical methods [18]. Semiquantitative immunohistochemical analysis revealed the general trend that the expression of RANK/RANKL/OPG was higher in human IVD tissues in an advanced stage of degeneration compared to that in an early stage of degeneration; these results correspond to the results of pro-inflammatory responses of human IVD cells in vitro in the present study. Therefore, the results of this study further support the hypothesis that the RANK/RANKL/OPG system plays a part in the pathogenesis of human IVD degeneration in the presence of a cytokines network.

To evaluate the effect of RANKL on the matrix metabolism (especially catabolic pathways) of human IVD cells, in the present study, the cells were cultured with RANKL in the presence or absence of proinflammatory stimuli (IL-1β). Similar to the results in our previous study using rat IVD cells [18], the administration of RANKL alone had no significant effect on the expression of catabolic factors, such as IL-1β and MMPs, by human AF and NP cells. However those expressions were significantly more accelerated by RANKL stimulation with proinflammatory stimuli (IL-1β) than those of IL-1β alone.

Komuro et al. [13] have previously reported that RANKL alone did not induce the activation of NF-kappa B and the expression of proinflammatory mediators, and concluded that RANKL alone has no effect to stimulate the catabolic factors that are relevant to the pathogenesis of osteoarthritis. For this reason, Kwan et al. [14] have pointed out the small proportion of RANK-positive cells in human articular chondrocytes. From the results of our and previous studies [13, 14, 25, 26], we speculate that proinflammatory cytokines, including IL-1β, may have the ability to enhance RANKL signaling by affecting the quantity and/or quality (isoforms) [25, 26] of RANK found in human IVD cells. In short, RANKL may have the potential to stimulate the expression of catabolic factors in the proinflammatory cytokines-rich environment of degenerated IVDs.

To evaluate the effect of OPG on the expression of catabolic factors, in this study human IVD cells were cultured with rhOPG with or without IL-1β stimulation. Previous studies by Komuro et al. [13], in which human articular chondrocytes were cultured with OPG, showed no significant effect of OPG on the collagenase activity of human chondrocytes; however, Kwan et al. [14] showed that the protein level of MMP-13 and PAR-2 was significantly upregulated by OPG-Fc (composed of the RANKL-binding domains of OPG linked to the Fc portion of IgG). Kadri A. et al. [27] reported in a murine osteoarthritis model that the systemic administration of OPG suppressed the expression of ADAMTS-4 and ADAMTS-5 by murine articular chondrocytes and prevented cartilage degradation in vivo. Thus, the effect of OPG on the induction of catabolic factors by articular chondrocytes has been controversial. The results of our study in human IVD cells demonstrated a general trend that the mRNA expression of catabolic factors stimulated by IL-1β was significantly suppressed by the administration of recombinant human OPG (rhOPG). This suggests that the addition of exogenous rhOPG would suppress RANKL signaling by interacting with the RANK-ligand that had been enhanced by IL-1β stimulation. Shimizu et al. [28] reported that the intra-articular administration of rhOPG prevented the progression of knee osteoarthritis in a murine model of osteoarthritis, supporting the inhibitory effect of OPG on the progression of matrix degradation in an inflammatory environment. The results of our and Shimizu’s [28] studies suggest the possibility that OPG could be applied for the treatment of human IVD degeneration by inhibiting RANKL signaling.

To further identify the inhibitory effect of RANKL signaling on the expression of catabolic factors by human IVD cells, the activity of RANKL was next specifically neutralized by anti-human RANKL monoclonal antibody (ahRANKL-mAb). The results of this study have shown, for the first time, that the ahRANKL-mAb has the potential to decrease the mRNA expression of catabolic factors upregulated by proinflammatory stimuli, including IL-1β. In addition, the quantity of MMP-3 released into the medium was also significantly suppressed by ahRANKL-mAb. These results also support our finding that RANKL has a potential role in regulating the expression of catabolic factors that are relevant to the pathogenesis of disc degeneration in the presence of proinflammatory stimuli.

It is now well known that a fully human monoclonal IgG2 antibody that specifically targets RANKL, ‘denosumab’, remarkably improves systemic bone mineral density in patients with osteoporosis [29]. Recent clinical studies have shown that ‘denosumab’ inhibited the progression of bone erosion in inflammatory diseases, such as rheumatoid arthritis [30, 31]. More, recently, anti-RANKL monoclonal antibody was systemically administered in a delayed-type hypersensitivity arthritis model; the treatment reduced the destruction of the subchondral bone and, in addition, serum levels of serum amyloid P component and MMP-3 [32]. Although these studies showed evidence that the systemic administration of anti-RANKL antibody inhibited bone destruction or erosion in inflammatory diseases or conditions, its inhibitory effect on cartilage degradation has not been proved [30–32].

Interestingly, Sato et al. [33] reported that the administration of anti-RANKL antibody to punctured rat intervertebral discs significantly downregulated the expression of proinflammatory cytokines, such as IL-6 and TNF-α, in dorsal root ganglion (DRG) neurons innervating the injured disc. Their results suggest that anti-RANKL antibody may have the potential to suppress the inflammatory response that is associated with pain transmission within the degenerated discs. The results of this study and Sato’s studies suggest that anti-RANKL antibody might have inhibitory effects against the inflammatory responses that are relevant to the progression of human IVD degeneration.

In 2008, Neogi et al. [34] reported in the secondary analysis of data from the fracture intervention trail (FIT) that the alendronate (ALN) treatment group showed lower spinal osteophyte and disc height narrowing scores than the placebo group, suggesting that the administration of bisphosphonate (ALN) would be responsible for the inhibitory effect on the progression of disc degeneration.

Luo and colleagues [35] evaluated the effect of ALN on lumbar IVD degeneration related to osteoporosis using an overiectomized (OVX) rat model. They reported that administration of alendronate in the OVX rat model significantly prevented cartilage endplate thickening, and improved histological scores of disc degeneration, suggesting that ALN treatment was effective in delaying the process of rat disc degeneration [35]. Thereafter, Song et al. [36] reported in the same animal model that the systemic administration of ALN inhibited disc height narrowing and the alterations of extracellular matrix and cellular components characterized in the degenerative IVD.

Considering these inhibitory effects of ALN on IVD degeneration in osteoporotic animal models, it can be speculated that anti-RANKL treatment may have a similar effect to suppress the progression of IVD degeneration in the patients with osteoporosis.

The inhibition of RANKL signaling would be more specific than the inhibition of IL-1β or TNF-α signaling on inflammatory reactions. Therefore, the authors speculate the possibility that RANKL inhibitors may have the advantage of reducing multiple adverse effects that have been identified by the clinical use of nonspecific immunosuppressive agents, such as TNF-α inhibitors [37, 38]. In addition, the anti-RANKL antibody, ‘denosumab’, has been clinically used for osteoporosis patients, and its safety [29–31] and cost-effectiveness compared to other osteoporosis treatments [39, 40] has been reported. Therefore, the clinical applicability of anti-RANKL antibody treatment for low back pain patients would be high.

There are several limitations in this study. First, all data were obtained from in vitro studies that mimic the in vivo conditions of human IVD degeneration. Second, human IVD samples with different MRI grades of disc degeneration were utilized in this study; therefore, a potential bias regarding the gene expression of RANK/RANKL/OPG and the response of IL-1β, RANKL, OPG or ahRANKL-mAb may exist. Third, this study focused on catabolic factors related to IVD degeneration, thus, further study is needed to evaluate anabolic factors to provide a detailed analysis of the mechanisms of disc degeneration. Fourth, although the effects of IL-1β, RANKL, OPG or ahRANKL-mAb were mainly evaluated by quantitative-PCR, additional evaluations of those effects by examining protein expression, such as by western blot, would further support the results of those mRNA expressions.

## Conclusions

In conclusion, the results of this study showed that the RANK/RANKL/OPG system exists (evaluated at both mRNA and protein levels) in AF and NP cells isolated from human IVD tissues, and that these levels were regulated by proinflammatory stimuli, such as that by IL-1β. In human AF and NP cells, treatment with RANKL, in the presence of proinflammatory stimuli, upregulated the expression of catabolic factors, including proinflammatory cytokines and matrix-degrading enzymes; this result was not seen with treatment with RANKL without proinflammatory stimuli. Therefore, the RANK/RANKL/OPG system would be included in the complex molecular mechanism of human IVD degeneration by interactions with the proinflammatory cytokines network. This is the first study to report that the administration of rhOPG or anti-RANKL antibody induced the downregulation of the expression of catabolic factors that had been upregulated by inflammatory stimuli by both human AF and NP cells. The results of these studies suggest the possibility that the inhibition of aberrant RANKL signaling in pathologic conditions would alleviate the progression of disc degeneration by downregulating catabolic factors, including proinflammatory cytokines and matrix-degrading enzymes, thus suggesting the potential use of OPG or monoclonal anti-RANKL antibody as a therapeutic agent against human IVD degeneration. It would also be of great importance in a future study to evaluate the effect of anti-RANKL antibody and/or rhOPG on the progression of IVD degeneration using an animal model in order to develop a novel treatment of IVD degeneration.

## Abbreviations

ADAMTS: A disintegrin and metalloprotease with thrombospondin motif
AF: Annulus fibrosus
ahRANKL-mAB: anti human RANKL mouse monoclonal antibody
ALN: alendronate
ANOVA: Analysis of variance
CEP: Cartilaginous endplate
DRG: Dorsal root ganglion
ELISA: Enzyme-linked immunosorbent assay
FBS: Fetal bovine serum
HE: Hematoxylin and eosin
HRP: Horseradish peroxidase
IgG: Immunoglobulin G
IL-1β: Interleukin-1 beta
IRB: Institutional Review Board
IVD: Intervertebral disc
MMP: Matrix metalloproteinase
MRI: Magnetic resonance imaging
NF-κB: Nuclear factor kappa B
NP: Nucleus pulposus
OPG: Osteoprotegerin
PCR: Polymerase chain reaction
RANK: Receptor activator of nuclear factor kappa B
RANKL: Receptor activator of nuclear factor kappa B ligand
rhIL-1β: Recombinant human interleukin-1 beta
rhOPG: Recombinant human osteoprotegerin
rhRANKL: Recombinant human receptor activator of nuclear factor kappa B ligand
rRNA: Ribosomal RNA
TNF-α: Tumor necrosis factor-alpha
TRAF: Tumor necrosis factor receptor-associated factor

## References

[1] Kepler CK, Ponnappan RK, Tannoury CA, Risbud MV, Anderson DG (2013). The molecular basis of intervertebral disc degeneration. Spine J.

[2] Millward-Sadler SJ, Costello PW, Freemont AJ, Hoyland JA (2009). Regulation of catabolic gene expression in normal and degenerate human intervertebral disc cells: implications for the pathogenesis of intervertebral disc degeneration. Arthritis Res Ther..

[3] Zhao CQ, Liu D, Li H, Jiang LS, Dai LY (2007). Interleukin-1beta enhances the effect of serum deprivation on rat annular cell apoptosis. Apoptosis..

[4] Vo NV, Hartman RA, Patil PR, Risbud MV, Kletsas D, Iatridis JC (2016). Molecular mechanisms of biological aging in intervertebral discs. J Orthop Res.

[5] Le Maitre CL, Freemont AJ, Hoyland JA (2005). The role of interleukin-1 in the pathogenesis of human intervertebral disc degeneration. Arthritis Res Ther..

[6] Le Maitre CL, Hoyland JA, Freemont AJ (2007). Catabolic cytokine expression in degenerate and herniated human intervertebral discs: IL-1beta and TNFalpha expression profile. Arthritis Res Ther..

[7] Cabal-Hierro L, Lazo PS (2012). Signal transduction by tumor necrosis factor receptors. Cell Signal.

[8] Johnson ZI, Schoepflin ZR, Choi H, Shapiro IM, Risbud MV. Disc in flames: roles of TNF-alpha and IL-1beta in intervertebral disc degeneration. Eur Cell Mater 2015;30:104–116; discussion 16-7. 10.22203/ecm.v030a08 PMC4751407 26388614

[9] Garcia-Cosamalon J, del Valle ME, Calavia MG, Garcia-Suarez O, Lopez-Muniz A, Otero J (2010). Intervertebral disc, sensory nerves and neurotrophins: who is who in discogenic pain?. J Anat.

[10] Luoma K, Vehmas T, Raininko R, Luukkonen R, Riihimaki H (2004). Lumbosacral transitional vertebra: relation to disc degeneration and low back pain. Spine (Phila Pa 1976).

[11] Anderson DM, Maraskovsky E, Billingsley WL, Dougall WC, Tometsko ME, Roux ER (1997). A homologue of the TNF receptor and its ligand enhance T-cell growth and dendritic-cell function. Nature..

[12] Boyle WJ, Simonet WS, Lacey DL (2003). Osteoclast differentiation and activation. Nature..

[13] Komuro H, Olee T, Kuhn K, Quach J, Brinson DC, Shikhman A (2001). The osteoprotegerin/receptor activator of nuclear factor kappaB/receptor activator of nuclear factor kappaB ligand system in cartilage. Arthritis Rheum.

[14] Kwan Tat S, Amiable N, Pelletier JP, Boileau C, Lajeunesse D, Duval N (2009). Modulation of OPG, RANK and RANKL by human chondrocytes and their implication during osteoarthritis. Rheumatology (Oxford).

[15] Gruber Helen E, Ingram Jane A, Hoelscher Gretchen L, Zinchenko Natalia, Norton H James, Hanley Edward N (2011). Constitutive expression of cathepsin K in the human intervertebral disc: new insight into disc extracellular matrix remodeling via cathepsin K and receptor activator of nuclear factor-κB ligand. Arthritis Research & Therapy.

[16] Mackiewicz Z, Salo J, Konttinen YT, Kaigle Holm A, Indahl A, Pajarinen J (2009). Receptor activator of nuclear factor kappa B ligand in an experimental intervertebral disc degeneration. Clin Exp Rheumatol.

[17] Rutges JP, Duit RA, Kummer JA, Oner FC, van Rijen MH, Verbout AJ (2010). Hypertrophic differentiation and calcification during intervertebral disc degeneration. Osteoarthr Cartil.

[18] Takegami N, Akeda K, Yamada J, Sano T, Murata K, Huang J (2017). RANK/RANKL/OPG system in the intervertebral disc. Arthritis Res Ther..

[19] Roughley PJ (2004). Biology of intervertebral disc aging and degeneration: involvement of the extracellular matrix. Spine (Phila Pa 1976).

[20] Byron CR, Barger AM, Stewart AA, Pondenis HC, Fan TM (2010). In vitro expression of receptor activator of nuclear factor-kappaB ligand and osteoprotegerin in cultured equine articular cells. Am J Vet Res.

[21] Gravallese EM, Manning C, Tsay A, Naito A, Pan C, Amento E (2000). Synovial tissue in rheumatoid arthritis is a source of osteoclast differentiation factor. Arthritis Rheum.

[22] Mladenovic Z, Saurel AS, Berenbaum F, Jacques C (2014). Potential role of hyaluronic acid on bone in osteoarthritis: matrix metalloproteinases, aggrecanases, and RANKL expression are partially prevented by hyaluronic acid in interleukin 1-stimulated osteoblasts. J Rheumatol.

[23] Takayanagi H (2007). Osteoimmunology: shared mechanisms and crosstalk between the immune and bone systems. Nat Rev Immunol.

[24] Steeve KT, Marc P, Sandrine T, Dominique H, Yannick F (2004). IL-6, RANKL, TNF-alpha/IL-1: interrelations in bone resorption pathophysiology. Cytokine Growth Factor Rev.

[25] Papanastasiou AD, Sirinian C, Kalofonos HP (2012). Identification of novel human receptor activator of nuclear factor-kB isoforms generated through alternative splicing: implications in breast cancer cell survival and migration. Breast Cancer Res.

[26] Sirinian C, Papanastasiou AD, Zarkadis IK, Kalofonos HP (2013). Alternative splicing generates a truncated isoform of human TNFRSF11A (RANK) with an altered capacity to activate NF-kappaB. Gene.

[27] Kadri A, Ea HK, Bazille C, Hannouche D, Liote F, Cohen-Solal ME (2008). Osteoprotegerin inhibits cartilage degradation through an effect on trabecular bone in murine experimental osteoarthritis. Arthritis Rheum.

[28] Shimizu S, Asou Y, Itoh S, Chung UI, Kawaguchi H, Shinomiya K (2007). Prevention of cartilage destruction with intraarticular osteoclastogenesis inhibitory factor/osteoprotegerin in a murine model of osteoarthritis. Arthritis Rheum.

[29] Bone HG, Bolognese MA, Yuen CK, Kendler DL, Wang H, Liu Y (2008). Effects of denosumab on bone mineral density and bone turnover in postmenopausal women. J Clin Endocrinol Metab.

[30] Cohen SB, Dore RK, Lane NE, Ory PA, Peterfy CG, Sharp JT (2008). Denosumab treatment effects on structural damage, bone mineral density, and bone turnover in rheumatoid arthritis: a twelve-month, multicenter, randomized, double-blind, placebo-controlled, phase II clinical trial. Arthritis Rheum.

[31] Deodhar A, Dore RK, Mandel D, Schechtman J, Shergy W, Trapp R (2010). Denosumab-mediated increase in hand bone mineral density associated with decreased progression of bone erosion in rheumatoid arthritis patients. Arthritis Care Res (Hoboken).

[32] Atkinson SM, Bleil J, Maier R, Kuhl AA, Thorn M, Serikawa K (2016). Anti-RANKL treatment inhibits erosive joint destruction and lowers inflammation but has no effect on bone formation in the delayed-type hypersensitivity arthritis (DTHA) model. Arthritis Res Ther..

[33] Sato M, Inage K, Sakuma Y, Sato J, Orita S, Yamauchi K (2015). Anti-RANKL antibodies decrease CGRP expression in dorsal root ganglion neurons innervating injured lumbar intervertebral discs in rats. Eur Spine J.

[34] Neogi T, Nevitt MC, Ensrud KE, Bauer D, Felson DT (2008). The effect of alendronate on progression of spinal osteophytes and disc-space narrowing. Ann Rheum Dis.

[35] Luo Y, Zhang L, Wang WY, Hu QF, Song HP, Su YL (2013). Alendronate retards the progression of lumbar intervertebral disc degeneration in ovariectomized rats. Bone..

[36] Song H, Luo Y, Wang W, Li S, Yang K, Dai M (2017). Effects of alendronate on lumbar intervertebral disc degeneration with bone loss in ovariectomized rats. Spine J.

[37] Patel D, Madani S, Patel S, Guglani L (2016). Review of pulmonary adverse effects of infliximab therapy in Crohn's disease. Expert Opin Drug Saf.

[38] Xue JB, Zhan XL, Wang WJ, Yan YG, Liu C (2016). OPG rs2073617 polymorphism is associated with upregulated OPG protein expression and an increased risk of intervertebral disc degeneration. Exp Ther Med.

[39] Jonsson B, Strom O, Eisman JA, Papaioannou A, Siris ES, Tosteson A (2011). Cost-effectiveness of Denosumab for the treatment of postmenopausal osteoporosis. Osteoporos Int.

[40] Silverman Stuart, Agodoa Irene, Kruse Morgan, Parthan Anju, Orwoll Eric (2015). Denosumab for Elderly Men with Osteoporosis: A Cost-Effectiveness Analysis from the US Payer Perspective. Journal of Osteoporosis.

